# Silver nanoparticle toxicity on *Artemia parthenogenetica* nauplii hatched on axenic tryptic soy agar solid medium

**DOI:** 10.1038/s41598-023-33626-w

**Published:** 2023-04-19

**Authors:** Minh Anh Do, Hoa Thi Dang, Nhinh Thi Doan, Hong Lam Thi Pham, Tuyet Anh Tran, Van Cam Thi Le, Tim Young, Dung Viet Le

**Affiliations:** 1 University of Science and Technology of Hanoi, Hanoi, Vietnam; 2 Faculty of Fisheries, Vietnam National University of Agriculture, Hanoi, Vietnam; 3 Aquaculture Biotechnology Research Group, Department of Environmental Science, School of Science, Faculty of Health and Environmental Sciences, Auckland University of Technology, Auckland, New Zealand

**Keywords:** Model invertebrates, Embryology, Metals, Assay systems, Microbiology, Environmental sciences, Biological models, Microbiology techniques, Nanobiotechnology

## Abstract

The use of gnobiotic brine shrimp (*Artemia* spp.) for ecotoxicology and bacteria-host interaction studies is common. However, requirements for axenic culture and matrix effects of seawater media can be an obstacle. Thus, we investigated the hatching ability of *Artemia* cysts on a novel sterile Tryptic Soy Agar (TSA) medium. Herein, we demonstrate for the first time that *Artemia* cysts can hatch on a solid medium without liquid, which offers practical advantages. We further optimized the culture conditions for temperature and salinity and assessed this culture system for toxicity screening of silver nanoparticles (AgNPs) across multiple biological endpoints. Results revealed that maxima hatching (90%) of embryos occurred at 28 °C and without addition of sodium chloride. When capsulated cysts were cultured on TSA solid medium *Artemia* were negatively impacted by AgNPs at 30–50 mgL^−1^ in terms of the embryo hatching ratio (47–51%), umbrella- to nauplii-stage transformation ratio (54–57%), and a reduction in nauplii-stage growth (60–85% of normal body length). At 50–100 mgL^−1^ AgNPs and higher, evidence of damage to lysosomal storage was recorded. At 500 mgL^−1^ AgNPs, development of the eye was inhibited and locomotory behavior impeded. Our study reveals that this new hatching method has applications in ecotoxicology studies and provides an efficient means to control axenic requirements to produce gnotobiotic brine shrimp.

## Introduction

Nanoparticles of noble metals, such as silver, exhibit distinct physical, chemical, and biological properties^1^. Silver nanoparticles (AgNPs) are well known for their anti-bacterial, anti-viral, and anti-fungal activities^2^. AgNPs are therefore widely used as constituents within textiles, food packing, cosmetics, detergents, and paints, with further applications in biomedicine and water purification^3, 4^. In the aquaculture industry, AgNPs are applied to improve water quality, as a functional feed additive, and for disease control^5^. However, the increasing application of AgNPs in consumer products and aquaculture leads to their inflow to aquatic environments which is of growing concern. The toxicity of AgNPs to numerous marine organisms has been reported, for example in brine shrimp (*Artemia salina*)^6, 7^, white leg shrimp (*Litopenaeus vannamei*)^8^, the Mediterranean mussel (*Mytilus galloprovincialis*)^9^, copepods (*Amphiascus tenuiremis*)^10^, and rainbow trout (*Oncorhynchus mykiss*)^11^. The behaviour of AgNPs in seawater is complex due to their dissolution into free silver ions (Ag^+^) and their variable aggregation level, which are influenced by the electrolyte composition of the media^12^. For example, AgNP toxicity to the marine medaka (*Oryzia melastigma*) is greater at higher salinities^13^, meanwhile, the bioaccumulation of AgNPs in clams (*Scrobicularia plana*) is greater at lower salinities^14^. AgNP toxicity also depends on their particle size and shape, concentration, and coating agent composition^15^. Different coating agents greatly influence the behavior, stability, and fate of AgNPs in various environments^16^. Hence, aqueous media such as seawater might be not ideal to study the toxicity of AgNPs due to many unknown interactions.

Another vital challenge in studying AgNP toxicity in vivo is the difficulty to eliminate unknown interactions between the host and microbial communities. Under conventional toxicology test conditions, the observed toxicity of AgNPs on brine shrimp (*Artemia* spp.) may in part reflect their influence on important host-associated microbial communities rather than solely through direct physiological impact on the host^17^. This problem can be addressed through the culture and use of gnotobiotic *Artemia* to eliminate complex effects of host-associated microbial communities on the host response to toxins, and to unravel important microbial influences^18^. However, contaminating sources of bacteria from the incubation seawater, air supply, and the cysts themselves require careful management.

To reduce microbial contamination, cysts must be decapsulated and disinfected with NaOH and NaOCl, neutralized with Na_2_S_2_O_3_, and washed with sterile seawater^19^. During incubation, the air supply must be filtered. Axenicity of *Artemia* nauplii are typically assessed on Marine Agar for five days, during which time toxicity screening has already begun or been completed. Although this protocol verifies the gnotobiotic condition of nauplii, when experiments are inadvertently initiated with non-sterile organisms prior to verification then inefficiencies are experienced since testing must be repeated^19^. This current culturing protocol is further disadvantaged since the effect of a tested compound on decapsulated cysts might also differ from that of intact cysts. Development of a more efficient method to produce gnotobiotic *Artemia* without a seawater medium and with a reduced risk of microbial contamination would thus offer a solution to these problems.

Tryptic Soy Agar (TSA) is a general-purpose medium for the isolation and cultivation of fastidious or non-fastidious microorganisms. Sterility testing for facility contamination typically requires an all-purpose medium such as TSA, which can support the growth of a broad range of bacteria and fungi^20^. Hence, TSA could potentially provide a novel solid medium for hatching Artemia whilst affording the added benefit of an in-situ monitoring system for microbial contamination. Without using seawater during incubation, the variation of AgNPs toxicity due to seawater’s variable ionic and dissolved organic composition could further be reduced^21^.

Given the potential of TSA as an effective incubation media to produce gnotobiotic *Artemia*, testing different conditions (e.g., salinity, temperature) during hatching is importantfor optimization and standardization. Herein, we aim to investigate whether Artemia cysts can hatch on solid TSA media and explore the possibility of using this novel technique for AgNP toxicity testing.

## Methods

### Artemia hatching preparation

**Figure 1:**
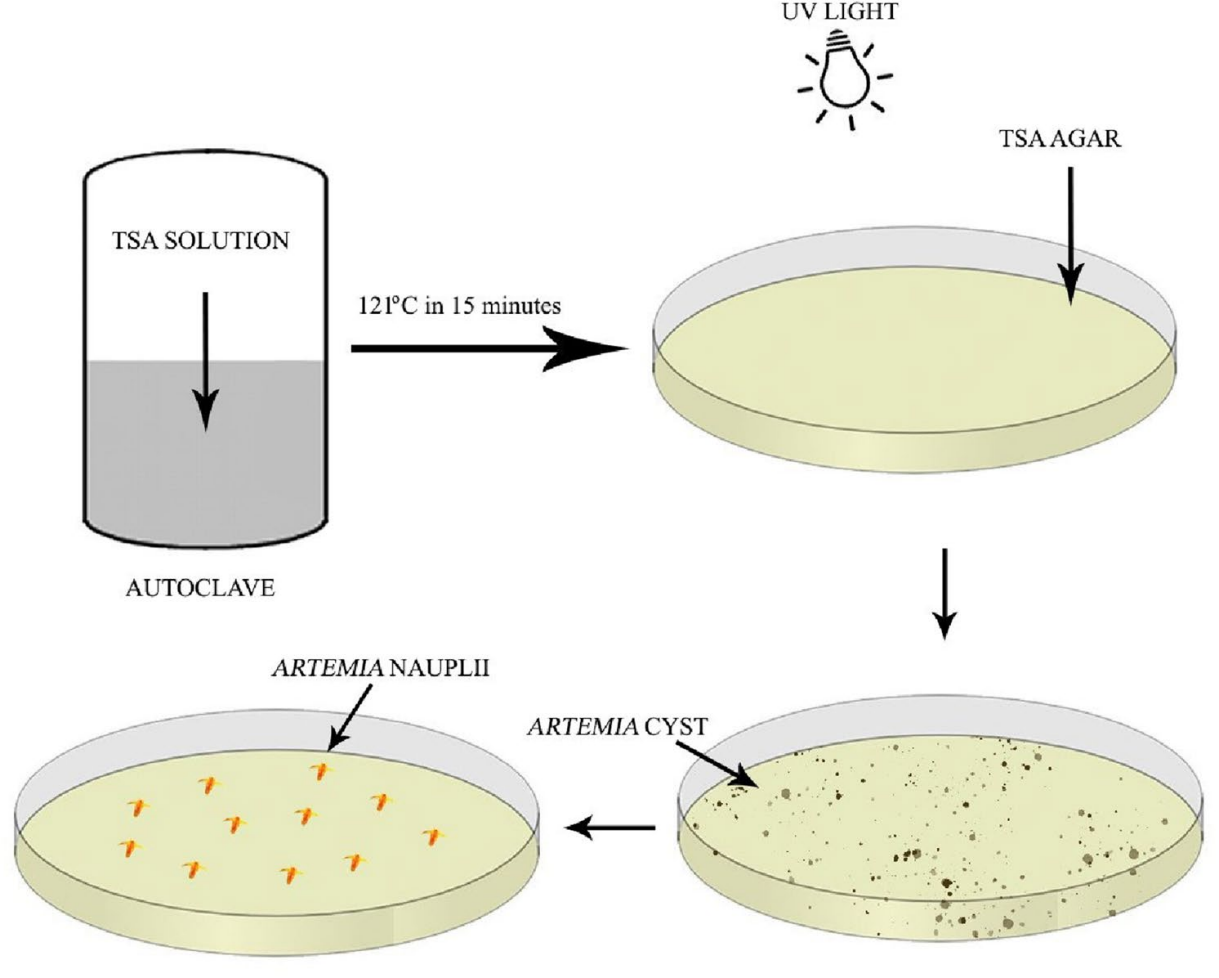
Tryptic soy agar (TSA) axenic environment used for hatching *Artemia* cysts.

*A. parthenogenetica* cysts were harvested in the Aral Sea (Uzbekistan) in 2020 and frozen for 24 months. After transfer to the MTT Joint Stock company (Vietnam) they were washed with brine water in a 50 L conical tank, and cysts which sank were collected on a 120 µm mesh sieve and excess water was removed. The moist cysts were air-dried at 38–40 °C within 8 h to decrease their moisture content below 8%. These cysts were stored in ziplock bags at 4℃ until used. To prepare the axenic solid medium for hatching cysts, 40 g of Trypic Soy Agar (TSA) powder (Merck, Germany) was prepared in 1 L of either distilled water or sodium chloride solution according to the experimental design below. The mixture was shaken for 5 min and boiled for the TSA to completely dissolve then sterilized via autoclave (121 °C; 15 min). After cooling to 45–50 °C, silver nanoparticles at different concentrations were added. 15 mL of these TSA stocks were spread evenly covering the surface of a Petri plate. TSA Petri plates were covered with the upper lid and subjected to UV light (55 W; 15 min) to maintain axenic conditions (Fig. 1). 0.001 g of *Artemia* cysts were sprinkled evenly on the surface of each TSA media. TSA plates were re-covered with the upper lid and with aluminum foil to avoid light exposure. All hatching procedures were conducted in a biosafety cabinet to minimize any bacterial contamination. All equipment were disinfected with 70% alcohol in the biosafety cabinet and UV-treated for 40 min prior to cyst preparation. There were 10 Petri plate replicates for each treatment unit.

### Silver nanoparticles

The AgNPs were obtained from Sil-Life™ (Taiwan) as a brown aqueous solution dissolved in pure water as the solvent. Properties of the AgNPs, as provided by the manufacturer, are displayed in Table 1.

**Table 1:** Characterization of silver nanoparticles used in this study.

Parameter	Analytical result	Analytical method	Note
Particle size	3.1–8.01 nm (x̄ 5 nm)	TEM Analysis	See SFig. 1
Composition	Silica	EDX Spectroscopy	See SFig. 2
Hydrodynamic diameter	8.5 nm	DLS Analysis	See SFig. 3
Homogeneity	Single peak at 405 nm	UV–Vis Spectroscopy	See SFig. 4
Concentration	10,203 mgL^−1^	ICP-OES (USEPA 2052)	
Halogen Fluorine	Not detected	IC (BS EN 14582: 2016)	
Halogen Chloride	Not detected		
Halogen Bromine	Not detected		
Halogen Iodine	Not detected		
Mercury (Hg)	Not detected	ICP-OES (IEC 62321-4:2013, AMD1:2017)	
Cadmium (Cd)	Not detected	ICP-OES (IEC 62321-5:2013)	
Lead (Pb)	Not detected		
Chromium VI (Cr^6+^)	Not detected	UV–Vis Spectroscopy (IEC 62321-7-2:2017)	
Polybrominated biphenyls (mono–deca)	Not detected	GC-MS + HPLC-DAD (IEC 62321-6: 2015)	
Polybrominated diphenyl ethers (mono–deca)	Not detected		

### Effect of temperature and salinity

To test the effect of temperature on *Artemia* hatching success, the TSA Petri plates with cysts were incubated at four temperature levels: 22, 25, 28 and 31 °C. The effect of salinity was investigated using four concentrations of sodium chloride, which were incorporated during the preparation of the TSA Petri plates: 10, 20, 30, 40 gL^−1^. Sodium chloride was dissolved in distilled water according to the treatment levels.

### Effect of AgNPs

The salinity and temperature levels which showed the best hatching performance in the first two experiments were selected to test the effect of AgNPs, using eight AgNP nominal concentrations: 30, 50, 100, 500, 1000, 2000, 3000 and 5000 mgL^−1^. A control was included without the addition of AgNPs.

### Hatching performance and morphological analysis

In the first two experiments (temperature and salinity effect) only the umbrella ratio was determined while in the third experiment the umbrella and the nauplii ratios were assessed. Both parameters were determined at 24 h and 48 h post incubation. Umbrella-stage individuals were counted if the cyst had burst and the embryo had appeared. Nauplii were counted if the hatching membrane had ruptured and the free-swimming form was present.

1. The percentage of umbrella-stage individuals:
  $${\text{H }} = {\text{ Number }}\,{\text{of }}\,{\text{umbrella}} - {\text{stage }}\,{\text{individuals/Total}}\,{\text{ cyst}}\,{\text{ on }}\,{\text{Petri }}\,{\text{dish }} \times { 1}00\%$$
2. The percentage of nauplii individual:
  $${\text{NI }} = {\text{ Number }}\,{\text{of}}\,{\text{ nauplii }}\,{\text{individuals/Number }}\,{\text{of }}\,{\text{umbrella}} - {\text{stage }}\,{\text{individuals }} \times { 1}00\%$$

The body length of ten random nauplii from each plate were measured under an inverted microscope (Nikon EL WD 0.3, Japan). The behavioral activity and morphology of those nauplii were also observed and described.

### Lysosomal damage

To determine lysosomal damage, the neutral red uptake (NRU) assay^22^ was applied with modification. This assay is based on the binding ability of viable cells in the nauplii to the supravital dye neutral red (3-amino-7-dimethylamino-2-methyl-phenazine hydrochloride) within lysosomes. Neutral red was dissolved in phosphate buffer solution (PBS) at 40 µg/mL then centrifuged at 600×*g* for 10 min to remove any precipitated crystals. The de-stain solution was prepared by mixing 50 mL ethanol (96%), 1 mL acetic acid and 49 mL deionized water. Ten nauplii at 48 h post incubation in the third experiment were pipetted into a 2 mL Eppendorf tube. 100 µL of the neutral red solution was added into each tube. The tubes were incubated for 2 h at 28 °C and then the dye solution was removed. Nauplii in each tube were washed with 150 µL PBS and then the PBS was removed. Next, 150 µL of the de-stain solution was added into each tube and gently shaken for 10 min to minimize the low affinity binding between neutral red and any untargeted substance. Lastly, those nauplii were transferred to Petri plates and observed under an inverted fluorescent microscope (Ex 525 nm; Em 645 nm).

### Statistical analysis

Hatching and nauplii percentage data were arcsine-transformed prior to statistical analysis to stabilize variances and normalize proportional data. Transformed data of hatching and nauplii percentage, and body length among treatments were compared using one-way analysis of variance (ANOVA) with pair-wise Tukey’s post hoc tests (α = 0.05) using SPSS software v.25.

## Results

### Effect of temperature

**Figure 2:**
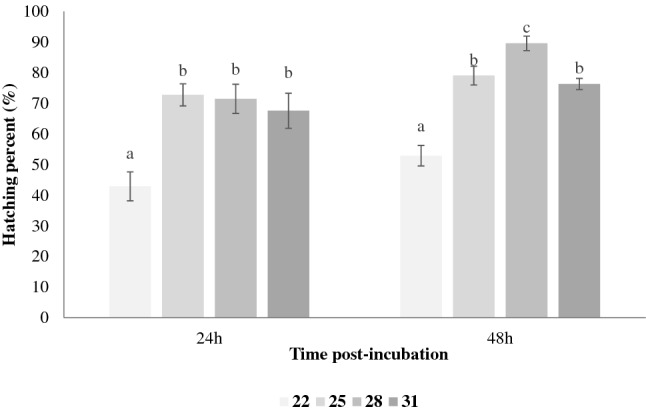
Effect of temperature on hatching percentage of *Artemia* cysts incubated on tryptic soy agar for 24 h and 48 h. Values are presented as means ± S.D. (n = 10). The same letters indicate statistically insignificant differences (*p* > 0.05).

Temperature influenced the hatching percentage of *Artemia* cysts on the TSA plates. The hatching ratios 24 h post incubation at 25, 28 and 31 °C were significantly higher (*p* < 0.05) than that at 22 °C (Fig. 2). However, the hatching ratios among the three higher temperatures showed no significant difference (*p* > 0.05). There was a clear unimodal trend of hatching dependence on temperature at 48 h post incubation (Fig. 2). The mean hatching percentage increased from around 52% at 22 °C to 90% at 28 °C, then declined to 76% at 31 °C (*p* < 0.05).

### Effect of salinity

**Figure 3:**
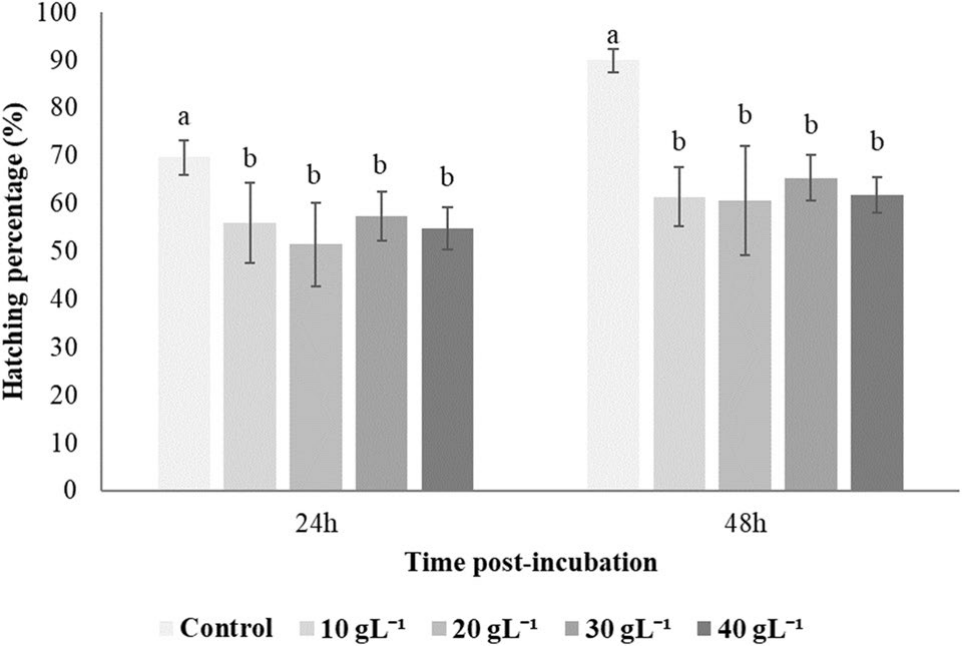
Effect of salinity on hatching percentage of Artemia cysts incubated on tryptic soy agar for 24 h and 48 h. Values are presented as means ± S.D. (n = 10). The same letters indicate statistically insignificant differences (*p* > 0.05).

The hatching ratios of *Artemia* cysts on the TSA plates were affected by the inclusion of sodium chloride in the media (Fig. 3). The hatching success 48 h post incubation decreased significantly (*p* < 0.05) from 90% in non-treated controls to around 60% at all levels of sodium chloride treatment. However, there was no difference in hatching ratios among sodium chloride treatments (*p* > 0.05). The same trend of hatching percentage was observed at 24 h post incubation.

### Effect of AgNPs

**Figure 4:**
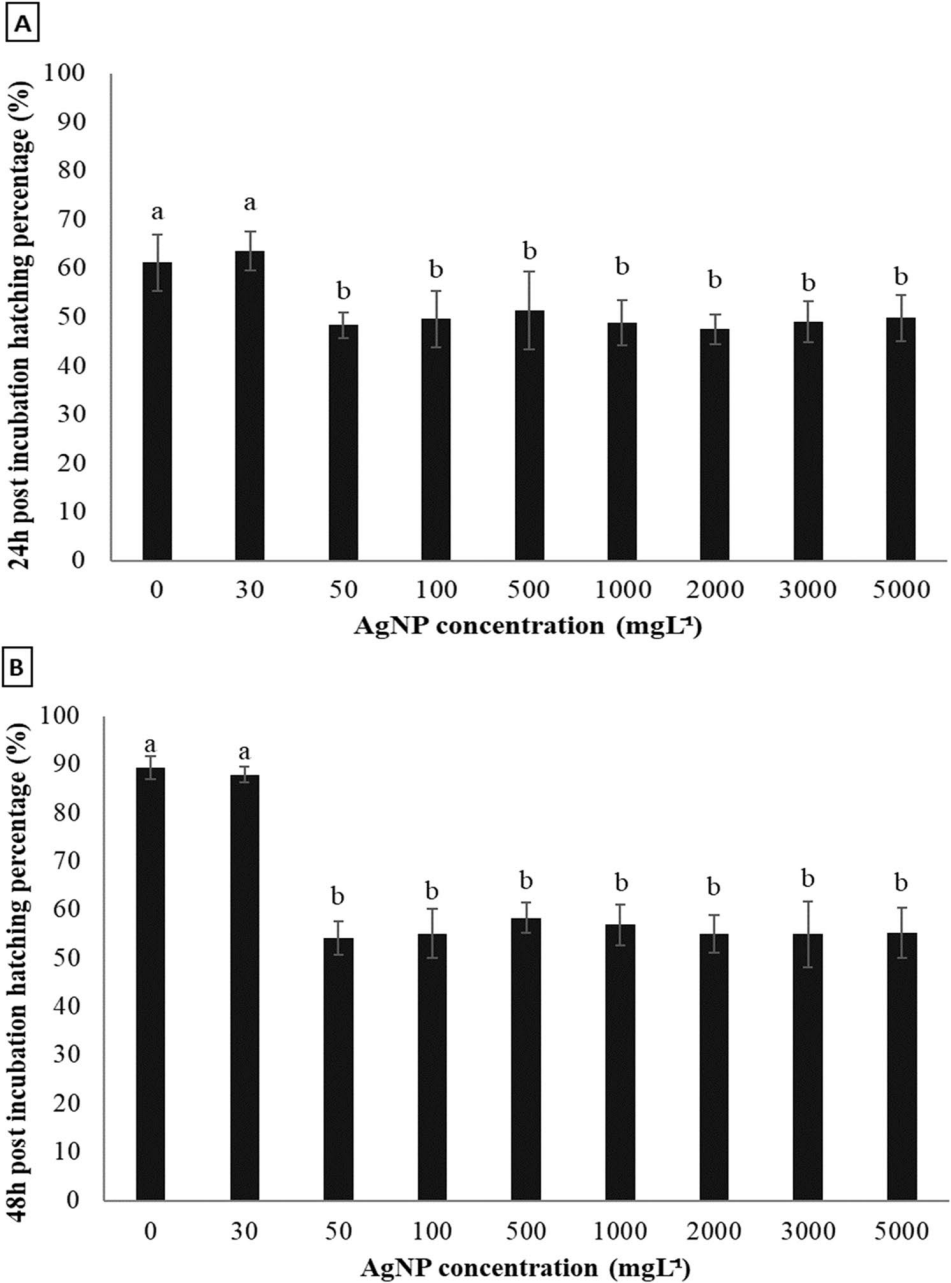
Effect of AgNP concentration on hatching percentage of Artemia cysts incubated on tryptic soy agar for 24 h **(a)** and 48 h **(b)**. Values are presented as means ± S.D. (n = 10). The same letters indicate statistically insignificant differences (*p* > 0.05).

**Figure 5:**
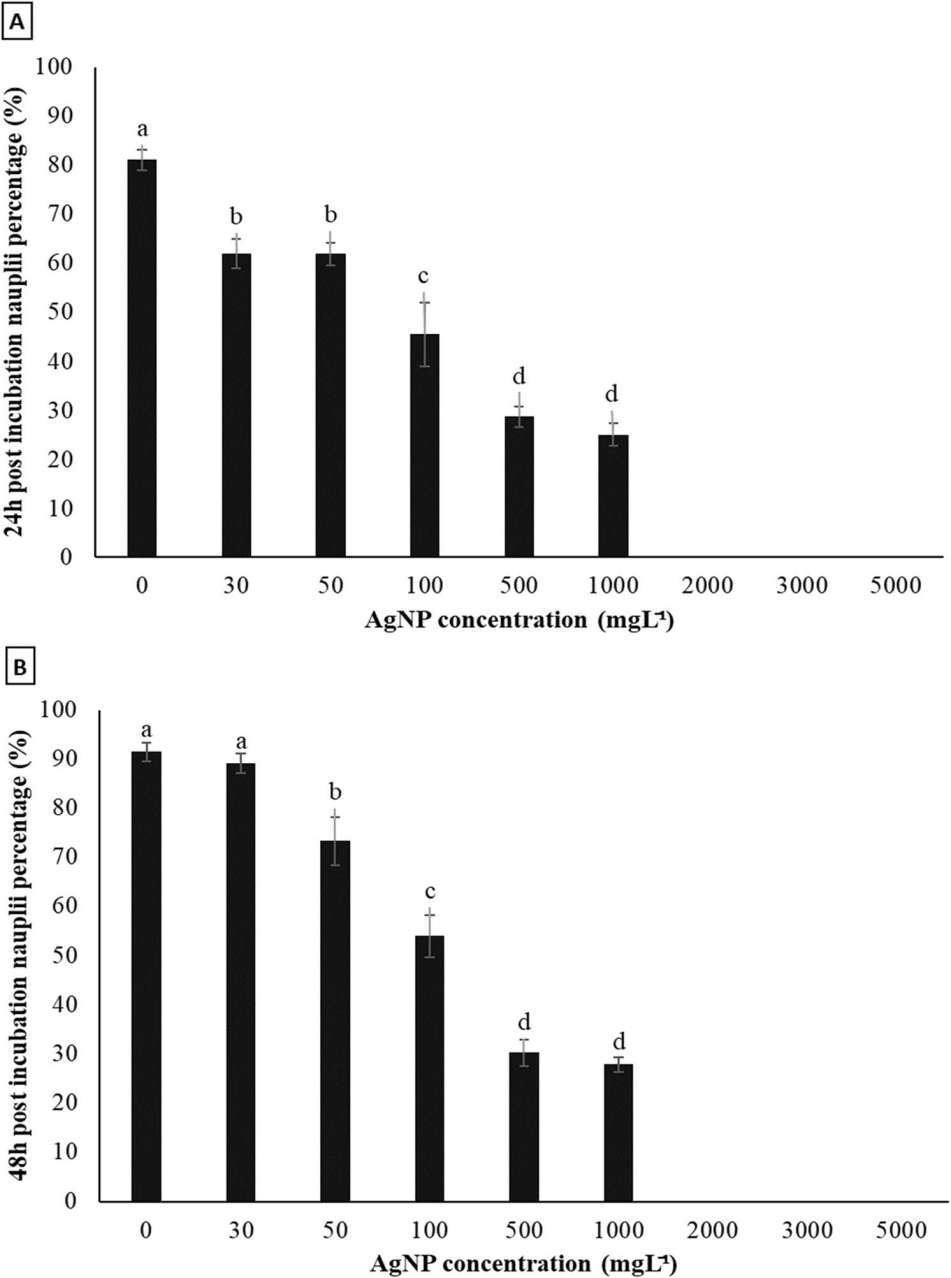
Effect of AgNP concentration on nauplii transformation percentage of Artemia incubated on tryptic soy agar for 24 h **(a)** and 48 h **(b)**. Values are presented as means ± S.D. (n = 10). The same letters indicate statistically insignificant differences (*p* > 0.05).

Inclusion of AgNPs in the TSA media resulted in a significant effect on both hatching and nauplii ratios at 24 h and 48 h post incubation (Figs. 4, 5). The hatching ratios at 24 h post incubation in the control and 30 mgL^−1^ AgNPs treatment were significantly higher (*p* < 0.05) than that in the other treatments (Fig. 4a). Interestingly, there was no difference in hatching ratios among 50–5000 mgL^−1^ AgNP treatments. A similar trend to 24 h post incubation observation was seen at 48 h post incubation. The hatching ratio in the control and 30 mgL^−1^ treatments achieved almost 90% and were higher than for other treatments (Fig. 4b).

In contrast, a stronger monotonically-based concentration effect of AgNPs was observed on nauplii stage transformation (*p* < 0.05) (Fig. 5). The nauplii percentage dropped from 80% in the control to 60% in the 30 and 50 mg L^−1^ treatments then to 27% in the 500 and 1000 mg L^−1^ treatments after 24 h (Fig. 5a). Although there was no difference in the nauplii ratio between the control and the 30 mg L^−1^ treatment after 48 h, there was a still clear trend of decreasing nauplii metamorphosis according to the increase of AgNPs inclusion (Fig. 5b). Noticeably, at ≥ 2000 mg L^−1^ AgNPs, nauplii were still absent after 48 h incubation.

### Morphological change

**Figure 6:**
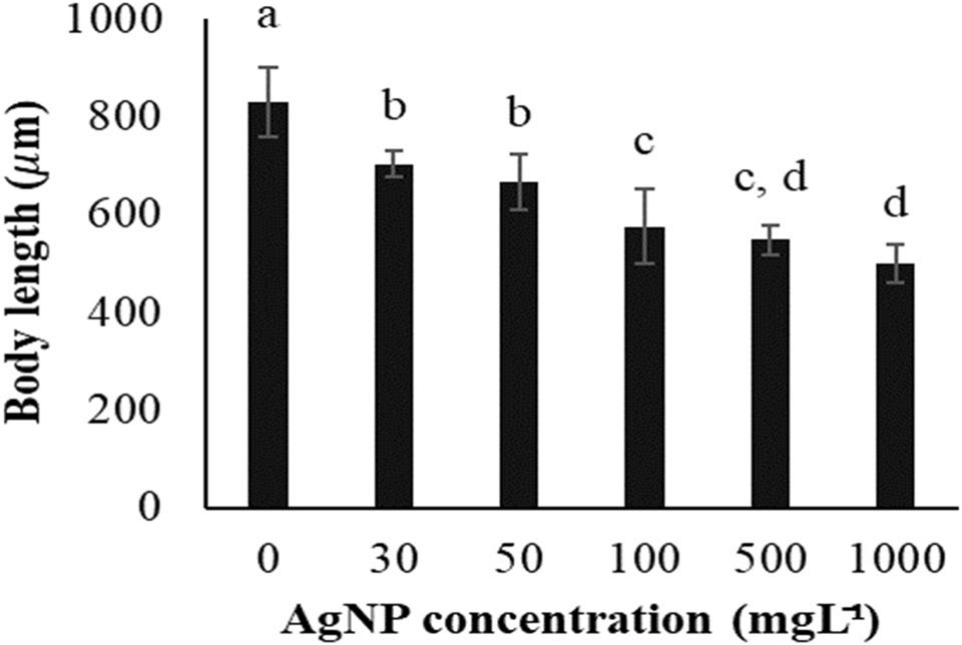
Effect of AgNP concentration on body length of Artemia nauplii incubated on tryptic soy agar for 48 h. Values are presented as means ± S.D. (n = 10). The same letters indicate statistically insignificant differences (*p* > 0.05).

**Figure 7:**
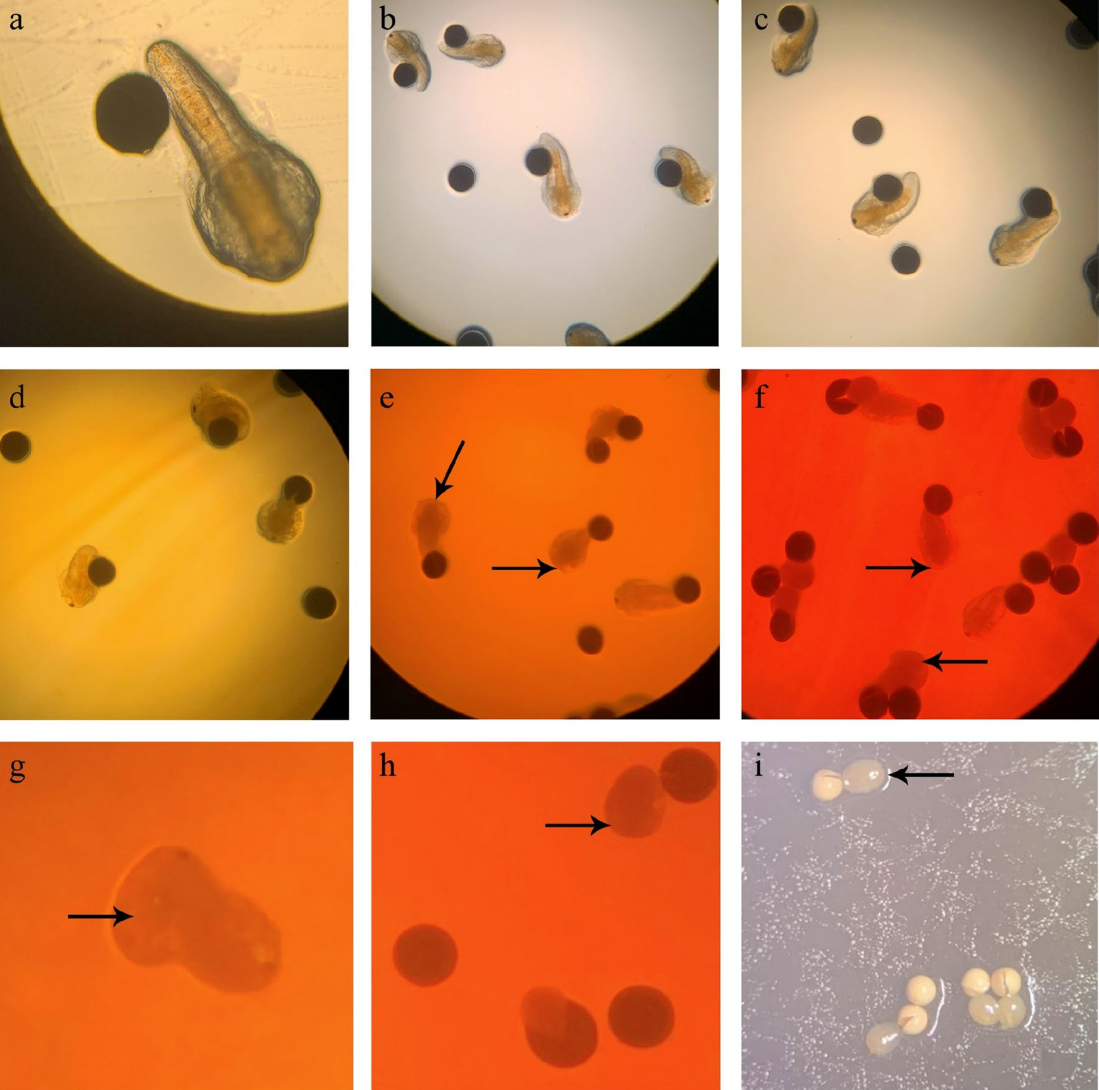
Morphology of *Artermia* in **(a)** control samples, **(b)** 30 mgL^−1^, **(c)** 50 mgL^−1^, **(d)** 100 mgL^−1^, **(e)** 500 mgL^−1^, **(f)** 1000 mgL^−1^, **(g)** 2000 mgL^−1^, **(h)** 3000 mgL^−1^ and **(i)** 5000 mgL^−1^ after 48 h of AgNP exposure. The arrows show the absence of eyes in samples **(e–f)**.

The body length (BL) of the nauplii was significantly (*p* < 0.05) affected by AgNP inclusion into the media. There was a decreasing trend in the BL with the increasing levels of AgNPs (Fig. 6). Control nauplii were the largest (BL ≈ 830 μm) while exposure to 1000 mgL^−1^ AgNP treatment resulted in substantial impact (BL ≈ 500 μm). The lowest AgNP dose tested (30 mgL^−1^) reduced (*p* < 0.05) nauplii BL to around 85% of their non-treated counterparts. Although BL was reduced, the appearance and behaviour of nauplii was otherwise normal up to 100 mgL^−1^ AgNP treatment (Fig. 7a–d). Those nauplii from the control, 30, 50, and 100 mgL^−1^ AgNP treatments were still actively swimming. Morphological and behavioural abnormalities (i.e., absence of eyes and weak movement) were observed at AgNP doses of 500 and 1000 mgL^−1^ (Fig. 7e,f). At higher AgNP concentrations no umbrella cysts succeeded in becoming nauplii (Fig. 7g–i).

### Lysosomal damage

**Figure 8:**
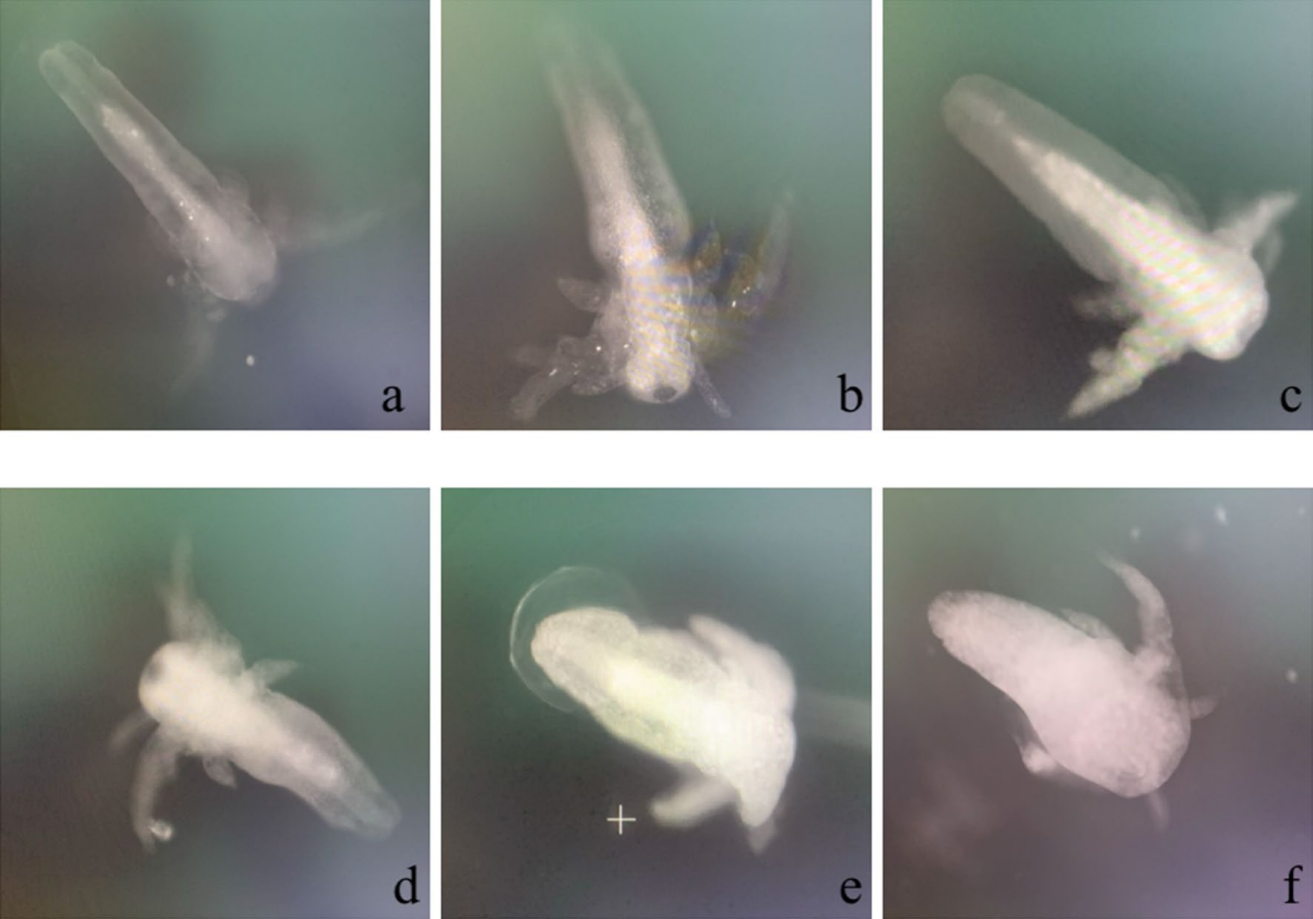
Lysosomal damage in *Artemia* nauplii in **(a)** control **(b)** 30 mgL^−1^, **(c)** 50 mgL^−1^, **(d)** 100 mgL^−1^, **(e)** 500 mgL^−1^, and **(f)** 1000 mgL^−1^ treatments’ samples. The individuals in control samples have a clear body and the lysosome increase gradually up to at 1000 mgL^−1^.

Although lysosomal damage was not quantitatively determined, there was a clear qualitative trend of increasing lysosome storage dysfunction with exposure of *Artemia* to increasing AgNP concentration (Fig. 8). In non-treated control animals, only a few bright white dots as lysosome were observed (Fig. 8a). The proportion of white increased to create a large volume in the nauplii under higher levels of AgNP treatment (Fig. 8b–f); especially at 1000 mgL^−1^ AgNPs the abnormal nauplii were full of lysosome (Fig. 8f).

## Discussion

The effect of temperature on the hatching of *Artemia* cysts on any solid medium (e.g., TSA) has not been reported to our knowledge. However, thermal effects on hatching and development of *Artemia* have substantially been studied in aqueous media. The highest hatching percentage of *A. franciscana* was reported at either 25 °C or 28 °C depending on the harvesting source^21, 23^. Meanwhile, the optimum temperature for *A. urmiana* was 30 °C^24^. In contrast, the hatching percentage was reduced with the increasing temperature levels from 25 to 30 °C for *Artemia* cysts from Great Salt Lake, USA^25^. With the solid TSA medium in our study, 22 °C decreased hatching percentages of *A. parthogenetica* cysts compared to other temperature levels. However, after 48 h, the optimum temperature in our study (i.e., 28 °C) was similar to that in previous studies^21, 26^.

The effect of salinity on hatching *Artemia* cysts incubated on TSA solid medium also has not reported to our knowledge. Although, like for temperature, previous findings have determined the effect of salinity in aqueous media^26, 27^. When testing with a wide range of salinity levels (15, 45, 60, 75, and 90 ppt) no difference was found in the hatching percentage of cysts^27^. In contrast, when experimenting with a narrow range of salinity levels the optimum level was determined at 28 ppt^26^. Surprisingly, on the TSA solid medium which contained 5 gL^−1^ of sodium chloride, the hatching percentage was the highest in this study. Adding more sodium chloride in the TSA medium caused reduction in hatching success. These observations differ from results of previous studies using aqueous media.

AgNPs are known to be toxic to *Artemia* spp*.* Most studies thus far have utilized newly hatched first instar nauplii at test organisms. The 48 h EC_50_ of AgNPs when exposed to hatched nauplii varies widely (ca. 20 to > 100 mgL^−1^) in interlaboratory studies based on the ‘immobilization assay’^28–30^. However, some earlier studies indicate that when using hydrated cysts as the initial test life stage then sensitivity to metals may be increased. The hatching success of decapsulated *A. franciscana* cysts decreased from 74% (control) to 32% after 24 h exposure to 10 mgL^−1^ AgNPs^31^. Here, we applied early exposure of non-decapsulated *Artemia* to AgNPs for the first time and assessed multiple toxicity end points (i.e., hatching and metamorphosis success, growth, eye development, and behaviour).

Results reveal that AgNP exhibited initial toxic effects at 30–50 mgL^−1^ under our test conditions, although not as severely as the previous report^31^ which may be due to capsulation and media effects. For example, apparent toxicity differences may stem from lower activation of Ag^+^ in the TSA solid medium compared with seawater. Surprisingly, hatching success was similarly affected by AgNPs from 50 to 5000 mgL^−1^. How cysts could resist such levels of AgNPs is uncertain. The cyst consists of five layers: the cuticular layer, alveolar layer, outer cuticular membrane, fibrous layer, and the inner cuticular membrane^32^. These layers serve as a filter to defend against environmental stressors and conserve the cell during the incubation process^33, 34^. During the hydration process of the dried cyst, the volume of the embryo can increase to a maximum of 140% water content^35^, providing a direct route for AgNPs/Ag^+^ into the embryo. Active metabolism initiates when cysts achieve around 60% water content and beyond^34^ and might be affected by AgNPs/Ag^+^ uptake during this time. However, after dried cysts completely change their form from biconcave to spherical, they cannot not absorb any more water, and presumably AgNPs/Ag^+^, which may play a part in why hatching success did not change among the 50–5000 mgL^−1^ AgNPs treatments. However, after 24 h there were no nauplii in the 2000–5000 mgL^−1^ AgNPs treatments. It is suggested that AgNPs and/or Ag^+^ could penetrate through the epidermis layer of the umbrella cysts and interfere with embryo development. Hence, AgNPs affected the artemia nauplii more severely than the cyst. Further work is needed to isolate the effect of Ag^+^, AgNPs, and coating substances.

This study showed that AgNPs cause *Artemia* nauplii abnormalities when incubated on solid media. Body length and the nauplii metamorphosis ratio were impacted at low AgNPs concentrations while the eye was completely under-developed at high AgNPs concentrations. When the nauplii were exposed to AgNPS in seawater abnormal nauplii were also observed^6^. Similar morphological changes such as deformation or developmental retardation of the eye, shrinking of the intestinal gut tract, degradation of the outer shell, as well as body length reduction were observed in *A. salina* nauplii exposed to ZnO NPs and TiO_2_ NPs^36^. Such nanoparticles might affect eye development in arthropods by disrupting endocrine-mediated processes and inhibiting ecdysteroid biosynthesis, similar to the mechanism of some organic pollutants such as bisphenol and sodium decocyl sulfate^37^.

Damage to lysosome storage was observed in nauplii even without obvious abnormal morphology after being exposed to lower AgNP levels and was extreme at higher doses. By increasing the intra-lysosomal pH, AgNPs interfered with lysosomal enzyme activity^38^. The increase of lysosomal storage may represent a compensation mechanism for impaired lysosome function caused by nanoparticles^39^. Lysosomal destabilization was also observed in AgNP-exposed hepatopancreas cells of oysters (*Crassostrea virginica*)^40^. Similarly, the number of lysosomes in the hepatocytes of rainbow trout (*Oncorhynchus mykiss*) exposed to AgNPs increased^41^. Lysosomes are known as vital intracellular organelles which are indicators for the cytotoxicity of nanomaterials^42^, hence, the dysfunction of lysosomal storage can be used for further toxicity assessment research in *Artemia* nauplii.

*Artemia* spp. are used extensively as test organisms in toxicology studies, however, for the shrimp to become an officially international recognized biological model in nanoecotoxicology further efforts are necessary^30^. Since *Artemia* cysts are typically incubated in liquid medium they are likely affected by variable aeration conditions, pH, seawater ion compositions, and microbial communities. For example, the toxicity results of heavy metals on the hatching success of *Artemia* was affected by Ca^2+^ and HCO^3−^ concentrations in the media^43, 44^. Hence, evaluation of hatching success is a difficult toxicity test to standardize with issues in reliability, reproducibility, and compatibility of toxicity data^45^. Our results reveal that a TSA solid medium is an applicable incubation technique which benefits from having a chemical composition that is easily controlled. Bioavailability of toxins within this medium is an area which requires further evaluation as this is currently unascertained. We envision further applications of this technique to facilitate the study of host-microbe interactions, mechanisms of innate immunity, nutritional requirements, and metabolic functions in *Artemia*^17^.

## Conclusion

Conventional methods to produce gnotobiotic *Artemia* in liquid media have practical limitations. We developed a novel solid media technique to hatch and culture Artemia which benefits from being more efficient and controllable than previous protocols. Cysts can hatch on TSA solid medium whilst exhibiting the axenic condition during the experimental period, and this technique also benefits from being able to monitor potential microbial contamination in-situ. Toxic effects of silver nanoparticles were evaluated on this medium to demonstrate applicability for ecotoxicological-based research, with further applications in host-microbe interaction studies being envisioned. Additional types of solid medium could be tested in future studies, and, although we have demonstrated proof of concept in this research, direct comparisons of liquid versus solid mediums in toxicity testing is advised.

## Supplementary Information

Supplementary Information.

## Data Availability

The data that support the findings of this study are available from the corresponding author, [LVD], upon reasonable request.
