# Supplementary material for: Silver nanoparticle toxicity on Artemia parthenogenetica nauplii hatched on axenic tryptic soy agar solid medium
Source: Sci Rep. 2023 Apr 19;13:6365. doi: 10.1038/s41598-023-33626-w (PMC10115835; doi:10.1038/s41598-023-33626-w)
Supplement: Supplementary file 1 — Supplementary Information. [file 41598_2023_33626_MOESM1_ESM.docx]

**Silver nanoparticle toxicity on *Artemia parthenogenetica* nauplii hatched on axenic tryptic soy agar solid medium**

Minh Anh Do^1^, Hoa Thi Dang^2^, Nhinh Thi Doan^2^, Hong Lam Thi Pham^2^, Tuyet Anh Tran^2^, Van Cam Thi Le^2^, Tim Young^3^, Dung Viet Le^2*^


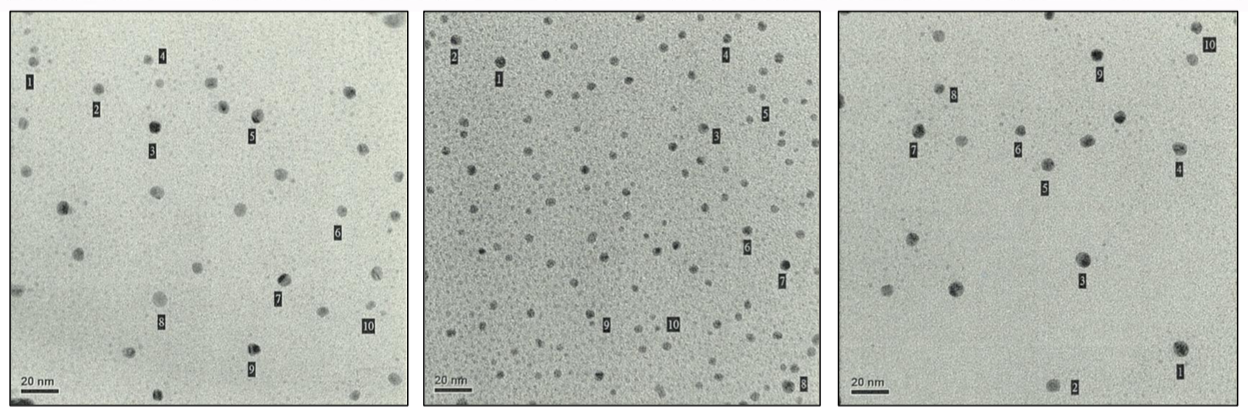
**Supplementary figures**


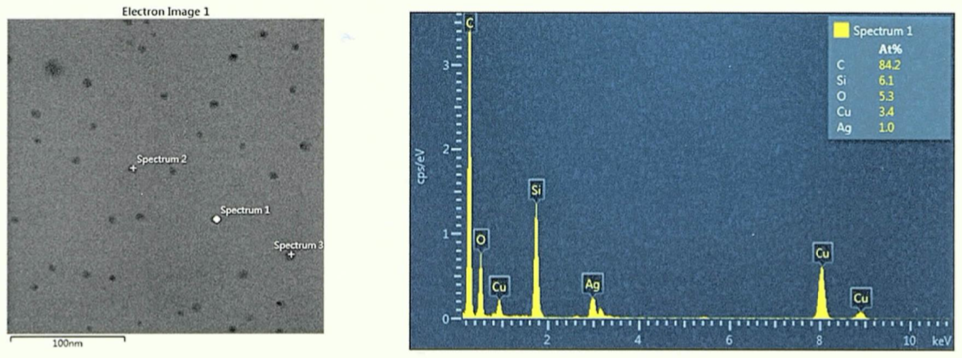
**SFigure 1** AgNPs characterized by TEM

**SFigure 2.** Total composition analysis by using X-RAY spectroscopy (EDS) on a TEM

**SFigure 3** Hydrodynamic size of AgNPs determined by Dynamic Light Scattering analysis

**SFigure 4.**UV-Vis spectroscopy results of AgNPs
